# Optimal channel and feature selection for automatic prediction of functional brain age of preterm infant based on EEG

**DOI:** 10.3389/fnins.2025.1517141

**Published:** 2025-01-28

**Authors:** Ling Li, Jiahui Li, Hui Wu, Yanping Zhao, Qinmei Liu, Hairong Zhang, Wei Xu

**Affiliations:** ^1^College of Communication Engineering, Jilin University, Changchun, Jilin, China; ^2^Department of Neonatology, The First Hospital of Jilin University, Changchun, Jilin, China

**Keywords:** preterm infants, functional brain age, EEG, channel selection, feature selection, SVR

## Abstract

**Introduction:**

Approximately 15 million premature infants are born each year, many of whom face risks of neurological impairments. Accurate assessment of brain maturity is crucial for timely intervention and treatment planning. Electroencephalography (EEG) is a noninvasive method commonly used for this purpose. However, using all channels and features for brain maturity assessment can lead to high computational burden and overfitting, which can decrease the performance of the prediction system.

**Methods:**

In this study, we propose an automatic prediction framework based on EEG to predict functional brain age (FBA) for assessing brain maturity in preterm infants. To optimize channel selection, we combine Binary Particle Swarm Optimization (BPSO) with Forward Addition (FA) and Backward Elimination (BE) methods. For feature selection, we combine the Pearson Correlation Coefficient (PCC), Recursive Feature Elimination (RFE), and Support Vector Regression (SVR) model.

**Results:**

The proposed framework achieved a prediction accuracy of 76.71% within ±1 week and 94.52% within ±2 weeks. Effective channel and feature selection significantly improved model performance while reducing computational costs.

**Discussion:**

These results demonstrate that optimizing channel and feature selection can enhance the performance of FBA prediction in preterm infants, offering a more efficient and accurate tool for brain maturity assessment.

## 1 Introduction

It is estimated that about 15 million babies (more than one in ten births) are born prematurely every year, and this number continues to rise (Blencowe et al., 2013). Premature birth often leads to neurological impairments in newborns, including immature brain development and varying degrees of brain injury (Gonzalez-Moreira et al., 2023). Additionally, the early immaturity state of the brain can have long-term effects on neurological development, learning abilities, and behavior during childhood (Ream and Lehwald, 2018; Rogers et al., 2018; Guarini et al., 2010). Effective monitoring of the brain functional maturity in the neonatal period enables timely intervention and the development of optimal treatment plans, improving neurodevelopmental outcomes for preterm infants. Brain functional maturity is reflected by the biological brain age that is functional brain age (FBA). Postmenstrual age (PMA) refers to the age since the mother’s last menstrual period when pregnancy began. The difference between the FBA and PMA, termed as brain age disparity, is a direct biomarker of brain functional maturity in preterm infants (Salih et al., 2023). In preterm infants with normal neurodevelopment, the PMA is the actual FBA. If the difference between them is more than 2 weeks, it indicates that the brain functions of premature infants have delayed or advanced development. Therefore, the accurate prediction of FBA in preterm infants is crucial for enhancing the assessment of neurodevelopment in clinical settings.

Neurological studies have demonstrated that electroencephalography (EEG) contains some markers of the brain functional maturity (Salih et al., 2023). EEG is a noninvasive method used to capture neuronal changes and display brain activity. It is widely utilized for early therapeutic decision-making and predicting neurodevelopmental outcomes in preterm infants. Currently, the primary method for assessing FBA in preterm infants relies on expert visual evaluation of the EEGs (Fogtmann et al., 2017). This approach supplements traditional anatomical measurements such as weight, length, and head circumference, and complements structural information from imaging techniques like cranial ultrasound and MRI (Twanow, 2017). Despite the convenience of EEG monitoring, raw EEG data are extensive and contain numerous artifacts, requiring experienced neurophysiologists to spend considerable time interpreting the data (Mathieson et al., 2016). The complexity of neonatal brain development further complicates the EEG evaluation, requiring years of experience for accurate interpretation, and subjective assessments can lead to inconsistencies among different physicians (Dempsey et al., 2018). Therefore, developing an automatic method to predict the FBA in preterm infants based on EEG is essential to improve the objectivity and quality of perinatal care.

With advancements in signal processing theories and machine learning technologies, researchers have attempted to extract features from EEG signals that reflect subtle changes in neurophysiological function and develop models to automatically predict the FBA of neonates. In these methods, FBA can be successfully predicted by training a regression model using the PMA as the label. Based on EEG, O’Toole et al. employed the spontaneous activity transients (SAT) detection algorithm (Palmu et al., 2010) to extract a linear combination of 41 amplitude, time, and spatial features and developed a support vector regression (SVR) model to estimate the FBA (O'Toole et al., 2016). The difference between the predicted FBA and PMA, that is prediction error, was 1.29 weeks for very preterm infants aged 23–32 weeks PMA. Stevenson et al. later applied this method to a broader PMA range of 24–38 weeks, achieving a prediction error of 1.49 weeks (Stevenson et al., 2017). They also compared data from two different hospitals and achieved a median prediction error of less than 1 week (Stevenson et al., 2020). Using the same feature extraction method, Dong et al. collected extensive EEG data from 1851 subjects and constructed a gradient boosting machine (GBM) model to predict the FBA, achieving a Pearson correlation coefficient (PCC) of 0.904 between the predicted FBA and PMA in normal neonates (Dong et al., 2021). De Wel et al. used visually marked quiet sleep (QS) periods and multiscale entropy features to predict the FBA using a linear regression model, resulting in a prediction error of 1.88 weeks (de Wel et al., 2017). Gschwandtner et al. extracted features from a convolutional neural network to assess the FBA, achieving a disparity of less than 2 weeks between the predicted FBA and PMA in 93.6% of cases (Gschwandtner et al., 2020).

Generally, the recorded EEG signals have several channels. If all channels are used in EEG analysis, some channels unrelated to the predictive goal may degrade model performance and efficiency. Therefore, channel selection is a key stage in the prediction of FBA of preterm infant like other EEG applications, such as depression detection (Shen et al., 2021; Zhang et al., 2022), seizure detection (Duun-Henriksen et al., 2012; Ra et al., 2021), emotion recognition (Yildirim et al., 2021; Javidan et al., 2021; Li et al., 2022), and brain-computer interfaces (Varsehi and Firoozabadi, 2021). In EEG channel selection, commonly used methods include filter, wrapper, and embedded approaches (Baig et al., 2020). Filter methods employ distance measures, statistical measures (Bhattacharyya and Pachori, 2017), and information measures (Qi et al., 2021), providing rapid responses and being classifier independent, though they often fall short in accuracy. Wrapper methods assess channels by training and testing classification algorithms, which can achieve higher accuracy but require more computational resources. Embedded methods can enhance performance by selecting channels during the classifier training phase and depending on specific classifiers. However, most studies on the prediction of FBA in preterm infants have not focused on channel selection, just like the methods mentioned above. Stevenson et al. reduced the number of channels due to the limitations of heterogeneity in electrode across two datasets and extracted features from two bipolar reference channels. Gschwandtner’s study explored 1, 4, and 8 bipolar reference channels, in which symmetric channel configurations were solely focused. However, this approach potentially overlooks other channel combinations that may yield higher accuracy (Gschwandtner et al., 2020). These attempts to reduce electrode channels are due to data limitations or clinical experience rather than optimal channels selection.

Another important stage of EEG based prediction of the FBA is crucial feature selection. Feature selection is essential for eliminating redundant features, improving model performance, and reducing overfitting. In predicting FBA in preterm infants, O’Toole et al. combined and compared features in various domains, such as the time domain, frequency domain, and a combination of both (O'Toole et al., 2016). However, this approach potentially overlooks the impact of individual features on model prediction accuracy. Stevenson et al. used feature label correlation and statistical tests to select features, which was prone to produce noise and often overlooked feature interactions (Stevenson et al., 2017). Dong et al. utilized a gradient boosting machine (GBM) with backward selection (Dong et al., 2021), however, this approach also overlooked the impact of feature interactions on the model’s performance. Overall, these methods lack a systematic approach in identifying the most relevant features for determining the FBA of preterm infants.

In order to better predict the FBA in preterm infants, we propose an automatic prediction framework based on EEG, in which we focus on optimizing channels and features selection. Our study is structured into four main parts. Firstly, we extract features from each channel across the amplitude, range-EEG (rEEG), inter-burst interval (IBI), spectral and nonlinear domains, respectively. Next, we propose a novel channel selection method by combining binary particle swarm optimization (BPSO) with forward addition (FA) and backward elimination (BE) methods. Then, in the feature selection phase, we utilize a feature selection method based on the combination of the PCC, recursive feature elimination (RFE) and SVR model, named PCC-RFE-SVR to enhance the performance prediction model. Lastly, in the model training and prediction phase, we train the most effective models using various regression techniques such as SVR, random forest (RF), gradient boosting decision trees (GBDT) and light gradient boosting machine (LGBM). By using fewer channels and features, the prediction accuracy and model’s portability are significantly improved, while reducing computational complexity and overfitting.

In a summary, the contributions of this study are as follows:

(1) Aiming to reduce unrelated channels and enhance the efficiency and accuracy in predicting the FBA in preterm infants, we propose a novel channel selection method that combines BPSO with FA and BE methods. It selects the optimal channels by minimizing the mean absolute error (MAE) between the predicted FBA and the actual FBA.
(2) In order to eliminate redundant features and consider the interactions of the features, we employ the PCC and RFE combined with the SVR method to select effective features from the selected channels.
(3) By optimizing the channels and features, we significantly reduce computational costs and overfitting, and enhance the model’s portability. Moreover, we train different models using advanced regression techniques such as SVR, RF, GBDT and LGBM to validate the performance of the proposed framework. The experimental results show that the proposed framework based on the SVR model provides the best performance for the EEG signals recorded by ourselves from the First Hospital of Jilin University, Norman Bethune.

The rest of this paper is organized as follows. Section 2 presents the materials and methods, including data description and preprocessing, feature extraction, feature normalization, evaluation metrics, channel selection, feature selection, and regression models. Section 3 presents the results of channels and features selection, hyperparameter optimization, performance comparisons, and prediction errors distribution analysis. Section 4 offers a discussion on the findings. Finally, the conclusion and future work directions are presented in Section 5.

## 2 Materials and methods

**Figure 1:**
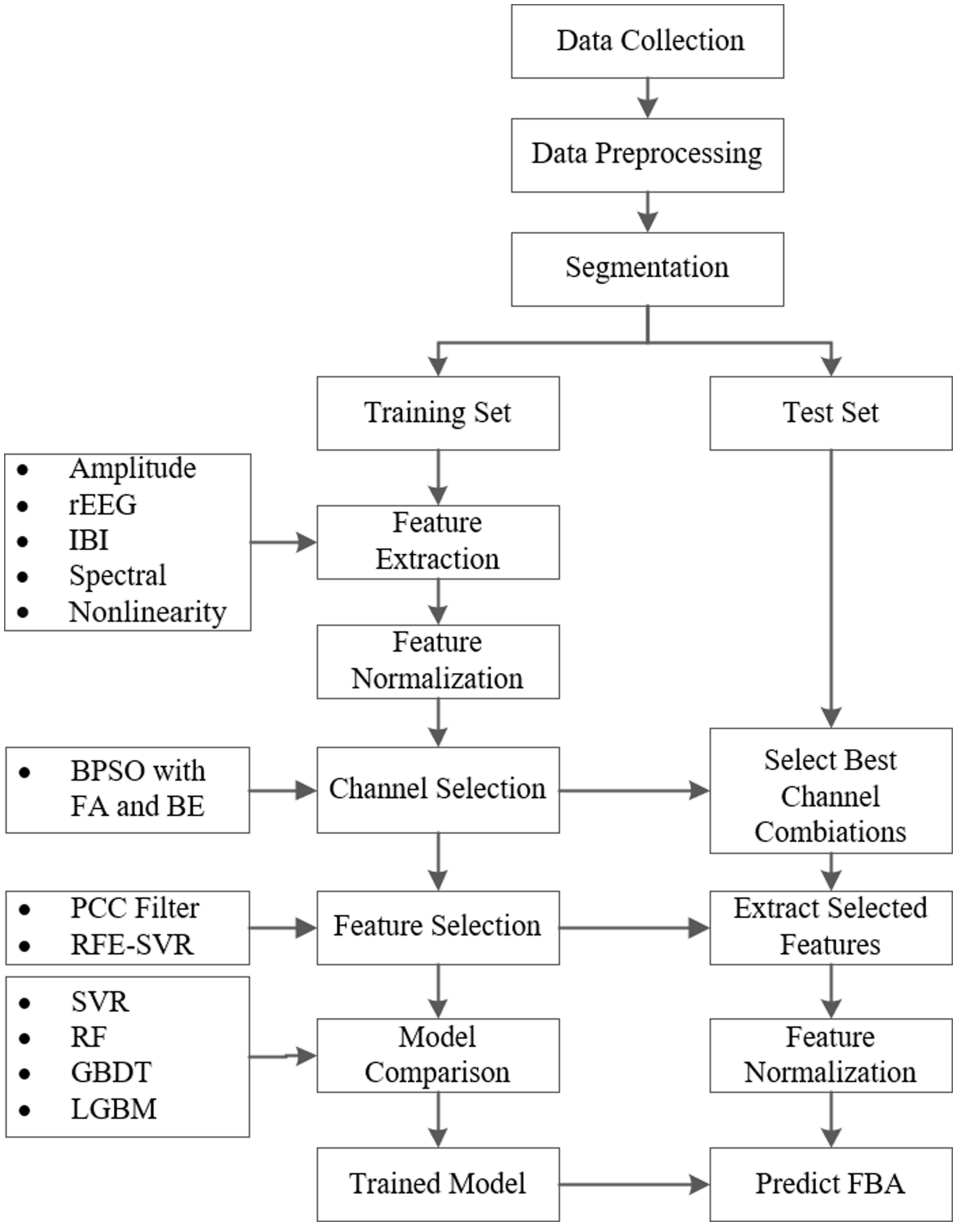
The implementation process of the proposed framework for the FBA prediction of preterm infants based on EEG.

The implementation process of the proposed framework for the FBA prediction of preterm infants based on EEG is shown in Figure 1. First, we collect EEG signals of preterm infants. After removing noise and artifacts by data preprocessing, we segment the preprocessed EEG data into one-hour intervals. Next, these data are divided into balanced training and testing sets across different age groups. Then we extract features from four frequency bands: delta (0.5–4 Hz), theta (4–7 Hz), alpha (7–13 Hz), and beta (13–30 Hz). We combine BPSO with FA and BE methods for channel selection. Following this, significant features are identified using the PCC filtering and RFE combined with the SVR model. Finally, to compare the performance of different models in predicting FBA, we train advanced regression models such as SVR, RF, GBDT, and LGBM. Subsequently, the trained models are then evaluated on the test set to ensure their generalizability and reliability.

### 2.1 Data description and preprocessing

**Figure 2:**
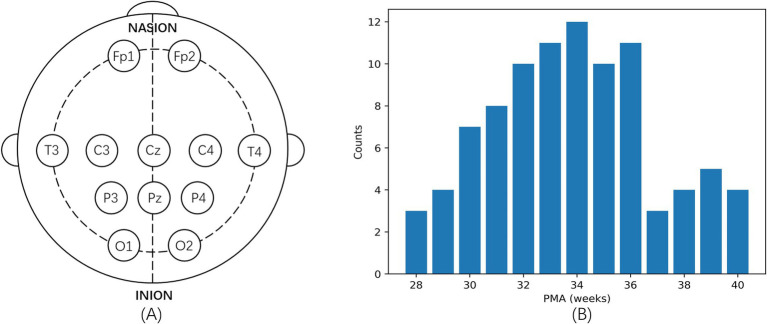
Overview of electrode placement and PMA distribution in the dataset. **(A)** Specific placement of electrodes. **(B)** Histogram of the PMA in the collected dataset.

From January 1, 2021, to December 31, 2022, we collected EEG signals of preterm infants treated in the neonatal intensive care unit of the First Hospital of Jilin University, Norman Bethune. Electrodes were placed according to the internationally modified neonatal 10–20 electrode placement system, with Cz as the reference electrode, and EEG signals were recorded using the Nicolet One EEG machine (Natus Medical Inc., Pleasanton, CA, United States) with 12 electrodes (Fp1, Fp2, T3, T4, C3, C4, P3, P4, O1, O2, Cz, and Pz). The specific placement of the electrodes is illustrated in Figure 2A. Each preterm infant was recorded only once, the sampling frequency was set at 500 Hz, with a duration ranging from 5 to 6 h. After the EEG signals were recorded, all identifiable information of the preterm infants was anonymized to ensure privacy, and a randomly generated unique identifier was assigned to each infant.

To ensure the accuracy and reliability of the data, we invited three expert doctors from Jilin University to interpret the EEG data. Based on the consensus reading of these experts, we select preterm infants with normal neurodevelopment as subjects for further study. Ultimately, we obtain EEG data from 92 preterm infants with normal brain development. Normal brain development means that the predicted FBA is consistent with the PMA, which is the actual FBA.

The dataset includes 53 male and 39 female preterm infants. The median birth weight is 1,650 g, ranging from 930 to 2,670 grams. The Apgar scores at 1 and 5 min have median values of 7 (5–9) and 8 (5–10), respectively. The median gestational age at birth is 32.04 weeks, with a range from 26.71 to 36.56 weeks. The median PMA is 34 weeks, with a range spanning from 28 to 40 weeks, as shown in Figure 2B, which displays a histogram illustrating the weekly distribution of infants within this range.

This study employs a bipolar reference method, specifically double banana longitudinal leads and symmetric bipolar references, resulting in a total of 16 bipolar channels. The names and corresponding IDs of these channels are detailed in Table 1. To reduce data complexity while preserving key information, the original EEG signals are first filtered using a 50 Hz notch filter to eliminate power line noise that may be introduced by the environment, and then resampled to 64 Hz. A fifth-order Butterworth filter is applied within the range of 0.5–30 Hz to remove unrelated frequency components. Subsequently, an artifact subspace reconstruction algorithm is employed to remove artifacts for further improving data quality (Chang et al., 2020). After these preprocessing steps, the artifact-free data are segmented into nonoverlapping one-hour segments for further analysis, resulting in a total of 485 one-hour EEG recordings.

**Table 1:** IDs and channel names for bipolar reference (ID is convenient for identifying the combination of channel selections below).

ID	Channel name	ID	Channel name
1	C3-C4	9	Fp2-T4
2	C3-P3	10	O1-O2
3	C4-P4	11	P3-O1
4	Cz-Pz	12	P3-P4
5	Fp1-C3	13	P4-O2
6	Fp1-Fp2	14	T3-O1
7	Fp1-T3	15	T3-T4
8	Fp2-C4	16	T4-O2

To ensure our model’s generalizability and reliability, we divide the data into training and testing sets within each age group in a 7:3 ratio. Importantly, this division is performed based on the unique identifiers assigned to the preterm infants after anonymization, ensuring that EEG data from the same infant does not simultaneously appear in both the training and testing sets. This approach prevents data leakage and maintains the independence of the test set while ensuring a balanced distribution between the two sets.

### 2.2 Feature extraction

To comprehensively capture the information contained in the EEGs of preterm infants, we reference the feature set proposed by Toole and Boylan (2017), where the features are extracted in the amplitude domain, rEEG domain, IBI domain, frequency domain, and nonlinear domain to obtain multidimensional EEG characteristics. In this study, each one-hour single-channel EEG signal is first divided into 119 epochs, using a sliding rectangular window of 60 s with a 50% overlap. Then each epoch is filtered into delta, theta, alpha, and beta frequency bands, respectively, by a fifth-order Butterworth filter, and specific features are then extracted from each frequency band. The average of the features from all epochs within the same frequency band is taken as the feature value for the corresponding frequency band and channel. Fractal dimension features are extracted at all frequencies. Unlike the feature set proposed by O'Toole et al. (2016), which is simplified by taking the median of features across all channels, this paper retains the features from all channels for subsequent channel selection.

#### 2.2.1 Amplitude domain features

In the amplitude domain, amplitude is quantified by signal power and mean envelope. Let $y\left[n\right]$ be the sample signal of the EEG signal of a channel, which is filtered into four frequency bands $y_{i}\left[n\right]$ , i=1,2,3,4 , corresponding to the delta, theta, alpha, and beta frequency bands, respectively, by a fifth-order Butterworth filter. The signal power $A_{i}^{\mathrm{power}}$ of the ith frequency band is defined as the average of the squared modulus of the signal over the number of sampling points N.

The envelope $e_{i}\left[n\right]$ of the ith frequency band is defined as using the modulus of the signal combined with the Hilbert transform, specifically $e_{i}\left[n\right]=|y_{i}\left[n\right]+jH\left\{y_{i}\left[n\right]\right\}|$ where $H\left\{\cdot \right\}$ represents the discrete Hilbert transform. The mean envelope $E_{i}^{\mathrm{mean}}$ is defined as the average of the envelope values over N sampling points. Additionally, the standard deviation, skewness, and kurtosis of $y_{i}\left[n\right]$ as well as the mean and standard deviation of the envelope $e_{i}\left[n\right]$ are extracted in this paper.

#### 2.2.2 rEEG domain features

The rEEG feature estimates a peak-to-peak measure of voltage by calculating the difference between the maximum and minimum values for adjacent intervals from the EEG signal, characterizing signal variability over time (O'Reilly et al., 2012). In this study, each epoch is segmented into 2 s with a 50% overlap. The peak-to-peak ranges are computed from all segments, and then feature metrics such as the mean, median, 5th and 95th percentiles (lower margin and upper margin), width (upper margin-lower margin), standard deviation (SD), coefficient of variation (CV), and measure of symmetry are derived from these ranges.

#### 2.2.3 IBI domain features

The IBI is a critical feature used to quantify the discontinuous bursting patterns observed in preterm infant EEGs. The IBI is defined as the interval between consecutive bursts of activity, with bursts identified using the burst-detection algorithm proposed by O'Toole et al. (2017). This method detects bursts by analyzing the amplitude of EEG signals, where high-amplitude activity is classified as a burst, and low-amplitude periods are classified as inter-burst intervals (Toole and Boylan, 2017).

To characterize the inter-burst pattern, several IBI-related features are extracted for each EEG channel, including the mean and median durations to reflect typical quiescent periods, the standard deviation and coefficient of variation to measure variability, and the burst ratio to capture the balance between bursts and quiescent periods.

#### 2.2.4 Spectral domain features

In the spectral domain, we first apply the Welch periodogram with a hamming window (2 s length, 50% overlap) to estimate the power spectral density (PSD) of the preprocessed EEG signals. Then we extract frequency domain features, including power in each frequency band obtained by short-time Fourier transform, relative power, Wiener entropy, Shannon entropy, differences between consecutive short-time spectral estimates, and spectral edge frequency (the cut-off frequency at which 95% of the spectral power is encompassed). The power in the spectral domain represents the integral of the PSD over the defined band’s frequency range, and relative power is the ratio of the power in a specific frequency band to the total power across all frequencies.

#### 2.2.5 Nonlinear features

For the brain’s complex and nonlinear nature, analyzing EEG signals from a nonlinear dynamics perspective may yield significant features that cannot be obtained by time domain and frequency domain analyses. The fractal dimension (FD) features, such as Petrosian, Katz, and Higuchi FD, are the nonlinear features (Esteller et al., 2001). In this paper, we apply the Higuchi method to extract the FD feature for each EEG channel, with the maximum interval between points in the time series of 6.

Table 2 summarizes the domains and specific names of features extracted from each channel, as well as the number of features extracted from each domain, where FB indicates whether the feature is extracted from each frequency band. In total, 86 features are extracted from all domains for each channel.

**Table 2:** The extracted features from EEG data (FB indicates whether the feature is extracted from each frequency band).

Domain	Features	FB	Number
Amplitude	Signal power, standard deviation, skewness, kurtosis, envelope mean value, envelope standard deviation.	Yes	4*6
rEEG	Mean, median, lower margin, upper margin, width, standard deviation, coefficient of variation, measure of symmetry.	Yes	4*8
IBI	Mean, median, standard deviation, coefficient of variation, burst ratio	No	5
Spectral	Power, relative power, Wiener entropy, Shannon entropy, differences between consecutive short-time spectral estimates, spectral edge frequency.	Yes	4*6
Nonlinearity	Fractal dimension	No	1

### 2.3 Feature normalization

The features extracted from different domains have various scales. To reduce the impact of these various scales on the machine learning model, we apply Z-score normalization to transform all feature data to follow a standard normal distribution. The normalization formula for a feature z is Equation 1.


(1) $$F=\frac{z-\mu }{\sigma }$$

where μ is the mean and σ is the standard deviation of the feature z. To ensure consistency and reliability, the 𝜇 and 𝜎 used for Z-score normalization of the test data are derived exclusively from the training data. To preserve the independence of each EEG channel and maintain the validity of subsequent channel selection, Z-score normalization is performed independently for each channel. This approach avoids inter-channel influence and ensures the integrity of the normalization process.

### 2.4 Evaluation metrics

Clinically, the allowable prediction error between the predicted FBA and the actual FBA (for normal preterm infants, the actual FBA is consistent with PMA) is defined as ±2 weeks for preterm infants (André et al., 2010). To evaluate the performance of the machine learning models in assessing the functional age of preterm infants, we use the MAE metric, which measures the average prediction error in weeks, providing a clear indication of how close the model’s FBA predictions are to the actual values. A smaller MAE indicates a better model performance. The MAE is defined as Equation 2.


(2) $$MAE=\frac{1}{K}\overset{K}{\underset{k=1}{\sum }}|Y_{k}-{\hat{Y}}_{k}|$$

where K is the number of samples, $Y_{k}$ is the actual FBA of sample k, and ${\hat{Y}}_{k}$ is the predicted FBA of sample k .

### 2.5 Channel selection

In the field of machine learning, it is a well-known fact that increasing the amount of data does not always correspond to an increase in effective information. This is particularly evident in the analysis of EEG data. Excessive channels may introduce more noise and redundancy, which may result in potentially degrading model performance and reducing its generalizability. Thus, channel selection becomes crucial for increasing the performance of the regression model. Its primary purpose is to identify and extract the most task-relevant channels, thereby reducing feature extraction time, lowering computational costs, avoiding data redundancy and enhancing model generalizability. This process not only helps improve model performance but also makes clinical testing more portable and operationally convenient (Alotaiby et al., 2015).

To effectively perform channel selection, we propose a novel approach by combining a multi-objective optimization algorithm with greedy algorithms, in which we use BPSO along with FA and BE methods to identify the optimal channels. The proposed method is based on SVR as the regression model, with the MAE as the performance evaluation metric. To ensure the accuracy and reliability of the evaluation, we employ 5-fold cross-validation on the training set.

BPSO is a multi-objective optimization method inspired by the social behavior of bird flocks, particularly in their collective search for food. BPSO is particularly suited for addressing discrete binary decision problems such as EEG channel selection (Lee et al., 2008). However, BPSO method may converge prematurely to local optima, particularly in complex search spaces, while it is finding a good initial solution effectively.

FA is a greedy algorithm used for channel selection and dimensionality reduction in EEG data analysis. It begins with an empty set and progressively adds channels, selecting the channel that maximizes a predetermined performance metric at each step, until a specified number of channels is reached or further additions no longer enhance the overall system performance. However, the FA method may be trapped in local optima and might not find the best global solution due to its myopic nature.

BE, similar to FA, is a greedy algorithm but it starts with the full set of channels. It progressively removes the least impactful channels based on a predetermined performance metric in each iteration, until the desired number of channels is maintained or further removals would significantly degrade the performance. Like FA, the BE method can also be limited by local optima and may not effectively explore the solution space to find the best global solution for channel selection.

BPSO method can quickly converge to the global optimal channel combination by exploring a wide search space, while FA and BE methods can optimize the performance metric further by adding or removing redundant channels. We combine BPSO with FA and BE methods to select optimal channels, which fully utilizes the global search capability of BPSO method and the fine-tuning precision of greedy algorithms. This combined approach provides several advantages, such as enhancing search capability through robust global and precise local search mechanisms, improving overall performance by ensuring both broad exploration and fine exploitation of the solution space, reducing redundancy, leading to lower computational costs and enhancing model efficiency. To ensure the independence of channel selection, feature selection is not applied during this process. Instead, the features from multiple channels are concatenated and directly fitted into the SVR model. The procedure of the proposed channel selection method by combining BPSO with FA and BE methods is described in detail in Algorithm 1.

#### ALGORITHM 1 The proposed channel selection method by combining BPSO with FA and BE algorithm

**Table:** 

1:	**Input**:
2:	Feature set of M channels $\left\{F_{Chl},F_{Ch2},\ldots ,F_{ChM}\right\}\text{.}$
3:	Number of particles: P
4:	Maximum number of iterations: L
5:	Learning factors $c_{1}$ and $c_{2}$ .
6:	Initial weight w .
7:	**Procedure:**
8:	**BPSO Initialization:**
9:	**Initialize population and velocity:**
10:	Initialize a population of P particles, where each particle is a binary vector of length M , 1 represents the
11:	selected channel and 0 represents the unselected channel.
12:	**For each** particle i and each dimension j :
13:	Initialize each position $x_{ij}\left(0\right)$ to 0 or 1 with equal probability.
14:	Initialize each velocity $v_{ij}\left(0\right)$ to a small random value between −1 and 1.
15:	**End For.**
16:	**Initialize** $p_{\mathrm{best}}$ **and** $g_{\mathrm{best}}$ :
17:	$p_{\mathrm{best}}$ (Personal best position): the best position (set of features) found by a particle so far. Initially set to
18:	each particle’s starting position and updated whenever a particle finds a new position with a lower MAE.
19:	$g_{\mathrm{best}}$ (Global best position): the best position found by any particle in the population so far. Initially
20:	set to the position of the particle with the lowest MAE among the initial positions.
21:	**Evaluate initial population:**
22:	**For each** particle i in the population:
23:	Decode the binary vector of the particle to identify the selected channels.
24:	Use the selected channel feature set to train the SVR model on the training data.
25:	Compute the MAE for the predicted FBAs by the SVR model.
26:	Store the computed MAE as the fitness value of the particle.
27:	**End For.**
28:	**BPSO Optimization:**
29:	**For each** iteration t from 1 to L :
30:	**For each** particle i :
31:	**For each** dimension j:
32:	Generate random numbers $r_{1}$ and $r_{2}$ from a uniform distribution in the range [0, 1].
33:	Update the velocity:
34:	$v_{ij}\left(t+1\right)=w\cdot v_{ij}\left(t\right)+c_{1}\cdot r_{1}\cdot \left(p_{ij}\left(t\right)-x_{ij}\left(t\right)\right)+c_{2}\cdot r_{2}\cdot \left(g_{j}\left(t\right)-x_{ij}\left(t\right)\right)$
35:	Update the position using the sigmoid function sigmoid $\left(v_{ij}\right)=\frac{1}{1+e^{-v_{ij}}}$ .
36:	$x_{ij}\left(t+1\right)=\{\begin{array}{ll} 1 & \mathrm{if random number}<\mathrm{sigmoid}\left(v_{ij}\left(t+1\right)\right)\\ 0 & \mathrm{otherwise} \end{array}$
37:	**End For.**
38:	**End For.**
39:	Evaluate the new positions’ performance using the SVR model and compute MAE.
40:	Update $p_{ij}\left(t\right)$ , $p_{\mathrm{best}}$ , $g_{j}\left(t\right)\;\mathrm{and}\;g_{\mathrm{best}}$ :
41:	**For each** particle i :
42:	**If** the MAE of particle i at position $x_{i}\left(t+1\right)$ < MAE of $p_{\mathrm{best},i}\left(t\right)$ :
43:	$p_{\mathrm{best},i}$ = $x_{i}\left(t+1\right)$
44:	**For each** dimension j :
45:	$p_{ij}\left(t+1\right)$ = $x_{ij}\left(t+1\right)$
46:	**End For.**
47:	**End If.**
48:	**For each** dimension j :
49:	**If** the MAE of $p_{ij}\left(t+1\right)$ < the MAE of $g_{j}\left(t\right)$ :
50:	$g_{j}\left(t+1\right)$ = $p_{ij}\left(t+1\right)$
51:	**End If.**
52:	**End For.**
53:	**If** the MAE of particle i at position $p_{\mathrm{best},i}$ < MAE of $g_{\mathrm{best}}$ :
54:	$g_{\mathrm{best}}$ = $p_{\mathrm{best},i}$
55:	**End If.**
56:	**End For.**
57:	**End For.**
58:	**Store optimal set:**
59:	At the end of each iteration, the current global best position $g_{\mathrm{best}}$ represents the best channel combination
60:	found in that iteration.
61:	Store the optimal channel combinations $S_{m}$ and its MAE.
62:	where m denotes the number of selected channels
63:	**Expand with FA and BE:**
64:	**Forward Addition:**
65:	Initialize $S_{fa}$ with selected channels $S_{m}$ from BPSO.
66:	**While** there are remaining channels to be added:
67:	Evaluate the addition of each remaining channel to $S_{fa}$ .
68:	Add the channel that results in the smallest MAE to $S_{fa}$ .
69:	Store the current best combination and its MAE.
70:	**End While.**
71:	**Backward Elimination**:
72:	Initialize $S_{\mathrm{be}}$ with selected channels $S_{m}$ from BPSO.
73:	**While** $S_{\mathrm{be}}$ has more than one channel:
74:	Evaluate the removal of each channel from $S_{\mathrm{be}}$ .
75:	Remove the channel that results in the best MAE from $S_{\mathrm{be}}$ .
76:	Store the current best combination and its MAE.
77:	**End While.**
78:	**Output**:
79:	The set of optimal channel set { $S_{1},S_{2},\ldots ,S_{M-1}$ } and their corresponding MAEs.

Additionally, we also utilize two other multi-objective optimization algorithms in place of BPSO to observe the final effects, namely genetic algorithm (GA) and simulated annealing (SA). GA is an optimization technique based on the principles of natural selection, which simulates the process of biological evolution through operations such as selection, crossover, and mutation, thereby iteratively improving the solution (Moctezuma and Molinas, 2020). SA, on the other hand, is a probabilistic optimization method that mimics the physical process of annealing. It explores the solution space in a high-energy state and accepts increases in cost randomly during the cooling process to avoid local optima, progressively refining toward the global optimum as the “temperature” gradually decreases (Jayakumar and Raju, 2010).

### 2.6 Feature selection

In the traditional machine learning models, all features are often considered equally important, which may lead to redundancy and reduce the generalization capability of the regression model. We employ a feature selection process by combining a PCC and RFE with an SVR model. This approach focuses on the correlations between features, effectively reducing redundancy and enhancing the model’s effectiveness.

The first step of the feature selection method used in this paper is to analyze the correlation between features using the PCC. The PCC is defined as Equation 3:


(3) $$PCC=\frac{\sum_{i=1}^{K}\left(F_{i}-\bar{F}\right)\left(Z_{i}-\bar{Z}\right)}{\sqrt{\sum_{i=1}^{K}{\left(F_{i}-\bar{F}\right)}^{2}\sum_{i=1}^{K}{\left(Z_{i}-\bar{Z}\right)}^{2}}}$$

where $F_{i}$ and $Z_{i}$ are the ith feature of the two kinds of different features respectively, $\bar{F}$ and $\bar{Z}$ are their respective means, and K is the number of samples. We compute the absolute PCC values for all feature pairs to identify the degree of linear relationship between them. Feature pairs with a high absolute PCC value (greater than 0.9) are considered highly correlated, indicating redundancy. In such cases, one kind of feature from each highly correlated pair is filtered out to ensure that only the most informative and nonredundant features are retained for further analysis.

Following the PCC filtering, we apply RFE and a SVR model as the estimator to select the significant features, which is named PCC-RFE-SVR. The RFE process involves iteratively fitting the model and removing the least important features based on the model’s coefficients until the optimal subset of features is identified. The process of the PCC-RFE-SVR method for feature selection is conducted as follows:

**Step 1.** Filtering: Use the PCC to filter out redundant features.

**Step 2.** Initialization: Begin with the set of features obtained after PCC filtering.

**Step 3.** Model Fitting: Fit the SVR model to the training data using the current set of features.

**Step 4.** Feature Ranking: Rank the remaining features based on their importance weights derived from the SVR model’s coefficients.

**Step 5.** Feature Elimination: Remove the least important feature based on the ranking.

**Step 6.** Iteration: Repeat steps 3 to 5 until the desired number of features is reached.

To determine the optimal features, we employ 5-fold cross-validation on the training set and evaluate the model’s performance using the MAE. This process helps in identifying the most relevant features while minimizing the risk of overfitting.

### 2.7 Regression models

To evaluate the performance of channel and feature selection, we initially employ SVR as the evaluation model. The SVR model is chosen due to its strong generalization ability and effectiveness in handling high-dimensional data, which aligns well with the nature of our dataset. Preliminary experiments also demonstrate that SVR performed well in this condition, justifying its use in the selection processes.

However, to further validate the effectiveness of the selected channels and features and to compare the overall performance of different regression models, we introduce three additional models: RF for its robustness to overfitting, GBDT for its flexibility in capturing nonlinear relationships, and LGBM for its computational efficiency and scalability. By comparing these models under the same channel and feature selection settings, we aim to comprehensively assess the relative effectiveness of each model.

## 3 Results

### 3.1 Equipment

This study is conducted on a Windows operating system, uses Python 3.9, and primarily utilizes the Scikit-learn and MNE libraries for data analysis and processing. All experiments are performed on a computer equipped with an Intel (R) Core (TM) i7-7700K CPU to ensure the smooth progress and reliability of the results.

### 3.2 Results of channel selection

Initially, an SVR model is used to train on the features extracted from each individual channel, and its performance is evaluated using the MAE of the predicted FBA in preterm infants. Subsequently, by integrating various optimization methods such as FA, BE, BPSO, SA, and GA with the SVR model, we identify the optimal channel combinations under different numbers of channels, and use the MAE to assess and compare the performance of these combinations. The parameters for the SVR model and the methods for channel selection used in the experiments are detailed in Table 3. The SVR model is optimized using grid search, where “np.arange” is a function from the NumPy library used to generate sequences of numbers. For example, np.arange (0.01, 1.1, 0.1) generates a sequence of the C_values starting at 0.01, ending before 1.1, with increments of 0.1. For the SA method, the parameters include the initial temperature (*T*), cooling rate (*α*), and number of iterations (*L*). For the GA method, the parameters include the population size (*PS*), number of generations (*NG*), mutation rate (*MR*), crossover rate (*CR*), and selection method (*SM*).

**Table 3:** Parameters required for channel selection methods.

Methods	Parameters
SVR	C_values = np.arange(0.01, 1.1, 0.1), gamma_values = [‘scale’, ‘auto’, 0.01, 0.1, 0.5, 1], kernels = [‘linear’, ‘rbf’, ‘poly’]
FA	The number of channel subsets added each time:1
BE	The number of channel subsets to delete each time:1
BPSO	P=100 , w=0.5 , $c_{1}=1.5$ , $c_{2}=1.5$ , L=100
SA	T=1.0 , α=0.8 , L=100
GA	PS=10 , NG=20 , MR=0.1 , CR=0.7 , SM = Probabilistic

#### 3.2.1 Performance of single channel

**Figure 3:**
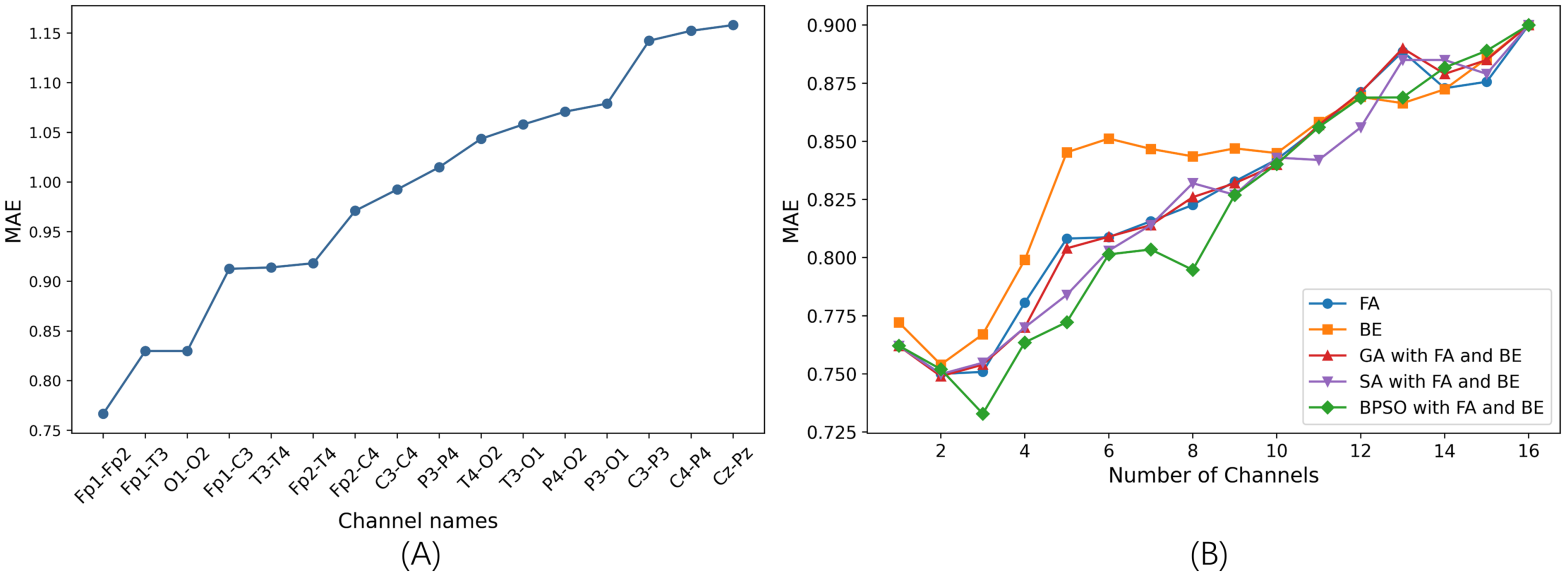
The results of channel selection based on 5-fold cross-validation on the training set. **(A)** Comparison of the MAE of different single channel based on SVR model (sorted in ascending order). **(B)** Comparison of the MAE between five channel selection methods based on the SVR model.

We train the SVR model on each of the 16 channels individually and optimize the model using grid search. The comparison of the MAE of different single channel is shown in Figure 3A. A smaller MAE indicates better regression model performance. In this study, the MAE represents the average prediction error of the FBAs of preterm infants in weeks. The results show that the Fp1-Fp2 channel achieves the best performance, with an MAE of 0.76 in 5-fold cross-validation. Conversely, the Cz-Pz channel has the poorest performance. Figure 3A clearly illustrates the significant differences in performance across different single-channel and shows that different channels have varying impacts on the final prediction results.

#### 3.2.2 Comparison of optimal channel selection methods

After determining the optimal single channel using the SVR model, next step is to find the best combinations of 2–15 channels. We employ proposed BPSO combined with FA and BE methods for channel selection, which is compared with other four channel selection methods: FA, BE, GA combined with FA and BE, and SA combined with FA and BE.

The comparison of the MAE is illustrated in Figure 3B. This figure shows the optimal results for different channel selection methods, with the x-axis representing the number of optimal channel combinations and the y-axis representing the MAE of the FBAs of these combinations in preterm infants. Notably, the MAE is lower when reducing the number of channels compared to using all channels (16 channels), indicating superior performance of channel selection methods. This demonstrates that effective channel selection can significantly improve prediction accuracy, as not all channels contribute positively to the accuracy of the model’s predictions.

Specifically, the BPSO with FA and BE method is notably effective in identifying optimal subsets of channels, achieving the lowest MAE of 0.73 with a three-channel combination. This highlights the critical importance of channel selection in enhancing the efficiency and performance of the model.

**Figure 4:**
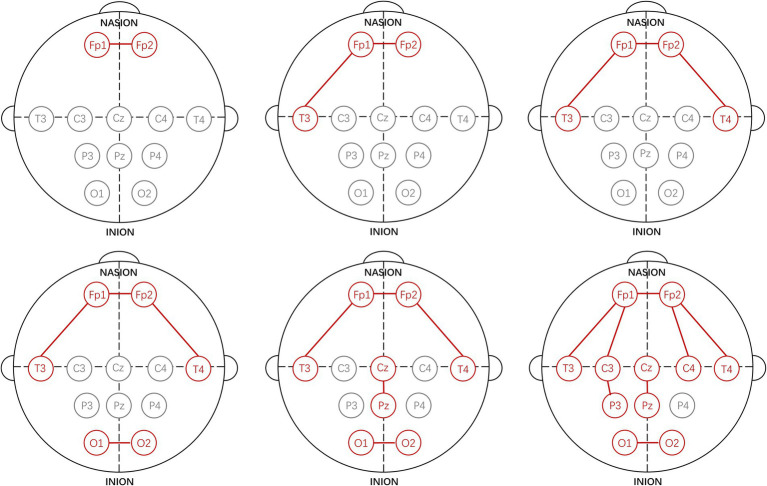
Specific electrode positions of the top six channel combinations with the minimum MAEs.

Using the proposed channel selection method, the top six channel combinations with the minimum MAEs are shown in Table 4. Table 4 indicates that the optimal performance is achieved using a three-channel combination (Fp1-Fp2, Fp1-T3, Fp2-T4), concentrated in the frontal and temporal lobes. Remarkably, the performance of the single-channel Fp1-Fp2 is also well with the MAE increasing by only 0.03 compared to the best three-channel combination, that is consistent with the result shown in Figure 3A. The specific electrode positions of the top six channel combinations with the minimum MAEs are detailed in Figure 4, where the red lines indicate bipolar references between the two electrodes.

**Table 4:** Top six channel combinations with the minimum MAEs.

Channel numbers	Channel sets (channel IDs)	MAE
1	6	0.76
2	6, 7	0.75
3	6, 7, 9	0.73
4	6, 7, 9, 10	0.76
5	4, 6, 7, 9, 10	0.77
8	2, 4, 5, 6, 7, 8, 9, 10	0.79

### 3.3 Results of feature selection

After obtaining the optimal three-channel combination by the proposed channel selection method, we use 86 features from each selected channel, resulting in a feature set with 258 features. In the feature selection phase, we employ the PCC-RFE-SVR method to identify the most appropriate features. Firstly, we remove 147 highly correlated features by the PCC filtering with a threshold of 0.9, so we obtain a feature set with 111 features. This step ensures the removal of redundant features that could negatively impact the model’s performance due to multicollinearity. Then, we apply RFE with the SVR model to further select the significant features. For comparison, we also apply two additional methods: PCC-SVR and RFE-SVR. The PCC-SVR method directly applies SVR after PCC filtering, without further feature refinement, while the RFE-SVR method skips the initial PCC filtering and directly applies RFE with SVR to the entire set of 258 features.

**Figure 5:**
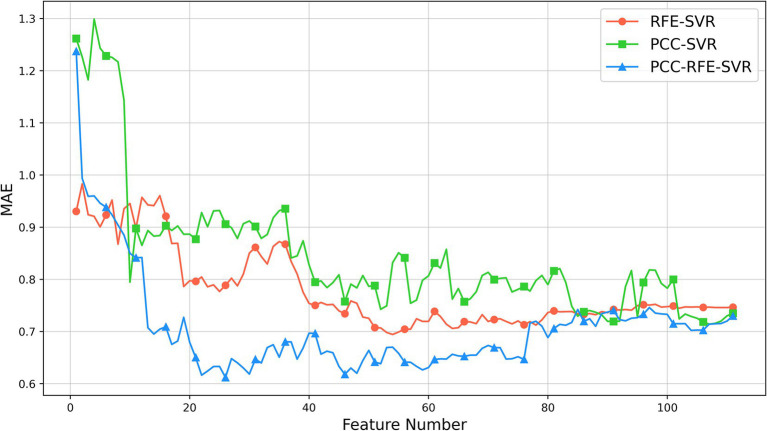
Results of feature selection based on 5-fold cross-validation on the training set.

The results of three feature selection methods are shown in Figure 5. As shown, initially, the MAEs of the three methods decrease as the number of features increases. As the number of features further increases, the MAEs of the PCC-SVR and REF-SVR methods change slowly without a clear minimum value, although the MAEs of the REF-SVR method are lower. However, the PCC-RFE-SVR method reaches its minimum value around 26 features. This optimal point represents the feature set where the model achieves the best performance with the lowest MAE, approximately 0.61. Beyond this point, adding more features leads to an increase in MAE, indicating that additional features can introduce noise and redundancy, thereby reducing the model’s performance.

Table 5 lists the selected features and $R^{2}$ values, sorted in descending order, where $R^{2}$ is the coefficient of determination, which is the square of the PCC. $R^{2}$ is always used to assess the performance of the feature selection method with larger value implying stronger positive correlation. The highest-ranked feature is “Lower margin” from the rEEG domain, located in the Fp2-T4 channel with theta frequency band (4–7 Hz). It has an $R^{2}$ value of 0.34, indicating a strong positive correlation with the FBA and making it the most significant contributor to improve the model’s performance.

**Table 5:** $R^{2}$ values of the 26 features selected by the PCC-RFE-SVR method.

Rank	Channel	Frequency band (Hz)	Domain	Feature name	$R^{2}$
1	Fp2-T4	Theta (4–7)	rEEG	Lower margin	0.34
2	Fp1-Fp2	Theta (4–7)	Spectral	Flatness	0.3
3	Fp1-Fp2	Alpha (7–13)	Amplitude	Skewness	0.3
4	Fp2-T4	Theta (4–7)	Spectral	Flatness	0.28
5	Fp2-T4	Alpha (7–13)	Amplitude	Skewness	0.27
6	Fp1-Fp2	Theta (4–7)	Amplitude	Kurtosis	0.27
7	Fp1-T3	Beta (13–30)	Amplitude	Kurtosis	0.23
8	Fp1-T3	Delta (0.5–4)	Amplitude	Kurtosis	0.21
9	Fp1-T3	Beta (13–30)	rEEG	CV	0.2
10	Fp1-Fp2	Beta (13–30)	rEEG	CV	0.19
11	Fp1-T3	Delta (0.5–4)	Amplitude	Skewness	0.17
12	Fp1-Fp2	Alpha (7–13)	Spectral	Flatness	0.15
13	Fp1-T3	Alpha (7–13)	rEEG	Asymmetry	0.12
14	Fp2-T4	Theta (4–7)	Spectral	Difference	0.12
15	Fp1-T3	All (0.5–30)	Nonlinear	FD	0.1
16	Fp1-T3	Beta (13–30)	rEEG	Lower margin	0.1
17	Fp1-Fp2	Beta (13–30)	rEEG	Asymmetry	0.07
18	Fp1-Fp2	All (0.5–30)	Nonlinear	FD	0.06
19	Fp1-T3	Alpha (7–13)	Amplitude	Kurtosis	0.06
20	Fp1-T3	All (0.5–30)	IBI	Burst ratio	0.05
21	Fp2-T4	Beta (13–30)	Amplitude	Envelope SD	0.04
22	Fp2-T4	Alpha (7–13)	Spectral	Power	0.02
23	Fp2-T4	Theta (4–7)	Spectral	Relative power	0.02
24	Fp1-T3	Beta (13–30)	Spectral	Power	0.01
25	Fp2-T4	Alpha (7–13)	Amplitude	Envelope SD	0.01
26	Fp1-Fp2	Alpha (7–13)	Spectral	Power	0.01

### 3.4 Performance of different regression models

The SVR model is chosen for its advantages in handling high-dimensional data and its strong generalization ability. To further assess the performance of SVR, we compare it with other regression models, including RF, GBDT, and LGBM. Consistency is ensured by using the same regression model for both channel selection and feature selection evaluation, with a 5-fold cross-validation on the training set.

As presented in Table 6, SVR consistently achieves the smallest MAE across all scenarios, regardless of the specific channel and feature selection methods or input configurations. This highlights SVR’s clear advantage in predicting FBA compared to RF, GBDT, and LGBM.

**Table 6:** Performance comparison of different models with channel and feature selection based on 5-fold cross-validation on the training set (CS, channel selection; FS, feature selection).

Model	Optimal channel combination	MAE without CS		MAE with CS	
		Without FS	With FS	Without FS	With FS
RF	6, 7	1.04	0.87	0.78	0.68
GBDT	6, 7	0.98	0.85	0.80	0.71
LGBM	6, 7, 9, 10	0.96	0.84	0.81	0.67
SVR	6, 7, 9	**0.90**	**0.74**	**0.73**	**0.61**

The bold values represent the optimal value in each column.

### 3.5 Performance comparison of channel selection and feature selection

In this study, we assess the proposed framework based on SVR using a feature set generated by concatenating features from different channels. For comparison, two additional cross-channel feature processing techniques, median and mean, are implemented. We evaluate these three methods median, mean and concatenation of processing cross-channel features using SVR, incorporating both channel and feature selection strategies, and apply 5-fold cross-validation on the training set to determine the optimal model, which is subsequently tested on the test set. The results, as shown in Table 7, highlight the outcomes across all experiments.

**Table 7:** Comparison of cross-channel feature processing, channel selection, and feature selection in the proposed framework.

Cross-channel feature processing	Channel selection	No. of channels	Feature selection	No. of features	MAE	
					Training set	Test set
Median	No	16	No	86	0.94	1.12
			Yes	12	0.79	0.95
	Yes	4	No	86	0.78	0.97
			Yes	18	0.69	0.82
Mean	No	16	No	86	0.95	1.21
			Yes	9	0.82	1.03
	Yes	2	No	86	0.81	1.02
			Yes	12	0.71	0.91
Concatenation	No	16	No	1,376	0.90	1.02
			Yes	279	0.74	0.85
	Yes	3	No	258	0.73	0.81
			**Yes**	**26**	**0.61**	**0.71**

The bold values represent the optimal value in each column.

Table 7 reveals that the performance of concatenation method outperforms those of the other two methods (median and mean) across various scenarios: with and without channel selection and feature selection. Specifically, for the concatenation method, channel selection alone reduces the MAE by 0.17 (from 0.90 to 0.73). Implementing feature selection in addition to channel selection further reduces the MAE by 0.12 (from 0.73 to 0.61). These results demonstrate that a combined approach of channel and feature selection using the concatenation method significantly lowers the MAE by a total of 0.29 (from 0.90 to 0.61), greatly enhancing model performance. Furthermore, the proposed framework with the concatenation method achieves an MAE of 0.71 on the test set, indicating good accuracy and generalizability of the established model.

### 3.6 Prediction errors distribution and statistical analysis on the test set

**Figure 6:**
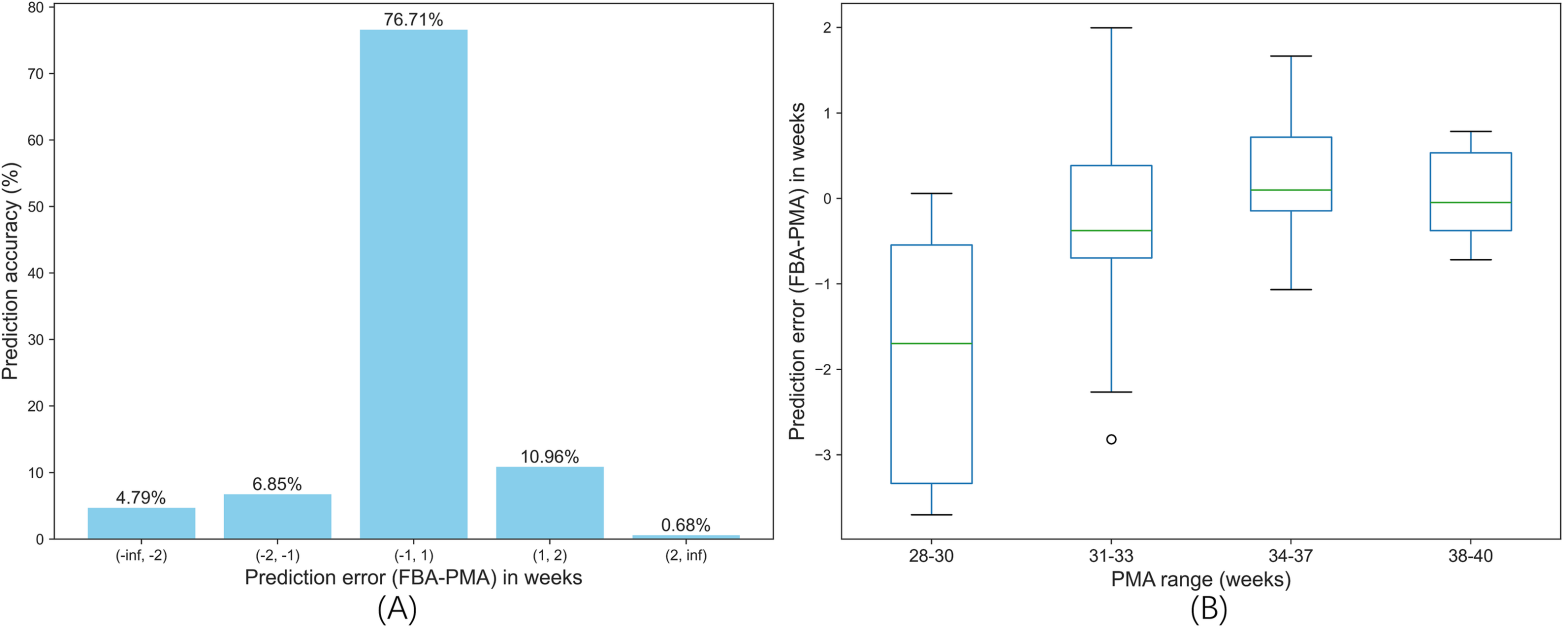
Error analysis of the proposed framework on the test set. **(A)** Prediction errors distribution of the proposed framework for predicting the FBAs of preterm infants. **(B)** Box plots of the prediction errors of different PMA Groups.

This section presents the distribution and statistical analysis of prediction errors on the test set using the trained optimal SVR model for predicting the FBAs of preterm infants. The prediction error is the difference of the predicted FBA and PMA (i.e., the actual FBA), the distribution is shown in Figure 6A, where the prediction errors are divided into five intervals: less than −2 weeks (from negative infinity to −2 weeks), −2 to −1 week, −1 to 1 week, 1 to 2 weeks, and greater than 2 weeks (from 2 weeks to infinity). The prediction accuracy of the proposed framework within ±1 week is 76.71%, and that within ±2 weeks is 94.52%.

Figure 6B illustrates box plots of the prediction errors of different PMA groups (28–30 weeks, 31–33 weeks, 34–37 weeks, and 38–40 weeks) of the entire dataset, where each box plot displays the distribution of prediction errors for each PMA group, including the minimum, first quartile (Q1), median (the middle green line), third quartile (Q3), and maximum values. As shown in Figure 6B, it is noted that the 28–30 weeks group has the largest range of prediction errors, with a median of about −2 weeks, indicating a tendency to underestimate the FMA. The larger prediction errors in this group might be due to a small sample size. The remaining PMA groups have smaller ranges of prediction errors, with medians close to zero, indicating more accurate FBA predictions for these PMA groups. The outlier in the 31–33 weeks, represented as circle, indicates the data point that lies more than 1.5 times the interquartile range (IQR) away from the first or third quartile. The outliers highlight individual prediction errors that significantly deviate from the general distribution for each PMA group.

## 4 Discussion

Nowadays, the automatic identification of EEG signals is being widely applied across various neurological domains, including the assessment of brain function maturity in preterm infants. To our knowledge, few studies focus on minimizing EEG channels specifically for predicting the FBA in preterm infants at present. Previous studies, such as those references (Stevenson et al., 2017; Gschwandtner et al., 2020), primarily concentrate on feature extraction. This study proposes an automatic prediction framework of the FBA in preterm infants with channel selection and feature selection based on SVR model for assessing brain function maturity, improving prediction accuracy compared to using all channels and features.

In the channel selection phase, we present a novel method that combines BPSO with two greedy algorithms, FA and BE. For BPSO method, while effective in finding a good initial solution, may converge prematurely to local optima, particularly in complex search spaces. FA method, as a greedy algorithm, may be trapped in local optima and cannot find the best global solution due to its myopic nature. Similarly, BE method can also be trapped in local optima and may not effectively explore the solution space for the best global solution. By combining these methods, we avoid these individual disadvantages and use their complementary strengths to achieve a more effective and efficient channel selection process.

The results indicate that the proposed channel selection method based on SVR model with fewer channels can achieve and even surpass the accuracy of using all channels. As shown in Table 4, among the various combinations explored, the three-channel combination of Fp1-Fp2, Fp1-T3, and Fp2-T4 provides the highest prediction accuracy. The MAE for the PMA and predicted FBA is 0.73 weeks under 5-fold cross-validation. The best single-channel is Fp1-Fp2, with an MAE of 0.76 weeks, whereas the full-channel combination has an MAE of 0.9 weeks.

However, the three channels positioned within the frontal and temporal regions are especially prone to artifacts due to muscle movements and eye blinks. Research has indicated that neonatal EEGs exhibit spatial heterogeneity in artifact manifestation across different channels (Webb et al., 2021). To enhance the objectivity of our EEG data analysis, we engage three clinical experts to review the data after artifact removal. Their expert examination confirms that our preprocessing significantly enhances the quality of EEG recordings, particularly in mitigating disturbances commonly found in the frontal and temporal lobes. This meticulous verification process ensures that the impact of artifacts is effectively minimized, affirming that neonatal brain development is intricately linked to activity in these regions (FP1, FP2, T3, and T4). Clinically, it suggests that focusing on these key channels can yield reliable diagnostic results, thereby enhancing the practicality and portability of EEG-based assessments of brain function maturity. Notably, we employ the ASR method for artifact removal, which is recognized for its high computational efficiency and prowess in identifying and removing high-variance artifacts. This method offers a substantial advantage over independent component analysis (ICA), which is computationally intensive and slow, necessitating considerable processing time and expert judgment to manage components effectively. Moving forward, we plan to explore more advanced and effective artifact removal techniques including those based on deep learning, to further refine EEG data analysis and improve the reliability of our findings.

During the feature selection stage, we combine the PCC, RFE and SVR model. The results of feature selection are as summarized in Table 5. We identify that the most critical features primarily originated from the rEEG and spectral domains, particularly within the theta (4–7 Hz) and alpha (7–13 Hz) frequency bands. The highest-ranked feature is the lower margin of the rEEG domain, which has a high $R^{2}$ value of 0.34, highlighting its significance in assessing brain activity. Spectral features such as flatness and skewness, which capture the nuances of brain activity, are also crucial, as are amplitude features like skewness and nonlinear features such as fractal dimension. These features ensure a comprehensive capture of various aspects of neonatal brain activity. The feature selection process significantly reduced the MAE of the FBAs, achieving the lowest MAE of approximately 0.61 with the optimal set of 26 features. This demonstrates the effectiveness of the PCC-RFE-SVR approach in enhancing model performance by removing redundant and less informative features.

Given that the EEG data in this study involves 16 channels, channel selection fundamentally represents a small-scale binary optimization problem (whether to select a certain channel). In this context, BPSO is a swarm intelligence-based algorithm capable of efficiently searching binary spaces to identify the optimal subset of channels. Its main advantage lies in its ability to escape local optima, making it well-suited for handling combinatorial problems. Furthermore, FA and BE can serve as enhancement techniques for BPSO by combining them to further augment the global search capability. As a result, the BPSO with FA and BE approach is appropriate for small-scale discrete optimization tasks, efficiently selecting the most informative channels from the 16 available. On the other hand, each channel’s feature set contains a substantial number of attributes (86 dimensions), among which significant correlations might exist. The goal of feature selection is to identify the features most correlative with the target variable while eliminating redundant or irrelevant features. To achieve this, the PCC quantifies the linear correlation between each feature and the target variable, rapidly discarding unrelated or weakly related features for initial screening. Subsequently, the PCC-RFE-SVR method further evaluates these features recursively on the basis of this preliminary screening. Given that the subsequent model optimization requires considering 86 features per channel, this feature selection process must balance efficiency and precision. Thus, it is designed to extract the most relevant features, thereby enhancing model performance.

Ultimately, both channel selection and feature selection are aimed at reducing dimensionality and minimizing redundant information, yet their purposes and the scales of the data they address differ. This divergence in objectives and data scale is pivotal in determining the choice of methods employed. In particular, channel selection focuses on reducing the number of EEG channels to handle, simplifying the initial data complexity, whereas feature selection refines the data further by extracting the most impactful features for accurate model predictions.

In various regression model tests, the SVR model consistently outperforms others in prediction accuracy, as shown in Table 6. As illustrated in Figure 6A, the SVR model achieves a prediction accuracy of 94.52% within a prediction error of ±2 weeks, and 76.71% within ±1 week in the test set. Clinically, the allowable prediction error between the predicted FBA and PMA is defined as ±2 weeks for preterm infants under 37 weeks PMA and ± 1 week for full-term infants. Significant errors indicate that the development of the brain functional maturity is delaying or advancing, necessitating clinical intervention (André et al., 2010).

This study’s EEG channel placements follow the internationally modified neonatal 10–20 electrode placement system. It is suggested that using three EEG channels can avoid some factors that influence prediction, such as noise, artifacts, and redundancy. These findings confirm the effectiveness of channel selection in the automatic assessment of brain function maturity in preterm infants. The small number of channels and concentrated feature set not only reduce computational costs but also enhance model portability, making it more suitable for a wider range of clinical applications. The proposed framework improves the objectivity and simplicity of EEG interpretation, potentially leading to better perinatal care outcomes.

Despite encouraging results, this study is constrained by the limited scale and imbalance of the dataset, which could impact the robustness and generalizability of the model. As shown in Figure 6B, the model performs poorly in segments such as the 28–30 weeks PMA group, where sparse data clearly affects performance. Although a larger and more diverse dataset would undoubtedly improve the model’s accuracy and generalizability, acquiring such a dataset is not straightforward due to constraints. In this context, data augmentation remains a powerful tool in machine learning that can help alleviate some of our challenges. To address this imbalance, we employ a combination of over-sampling and under-sampling techniques, along with the synthetic minority over-sampling technique (SMOTE). Specifically, SMOTE is used to artificially augment the dataset by creating synthetic samples, rather than over-sampling with replacement, which is a standard approach to managing imbalances in classification problems. We categorize preterm infants’ data by individual weeks or by combining adjacent weeks, then perform over-sampling or under-sampling on the extracted feature sets accordingly. However, despite these efforts, the use of SMOTE and other sampling strategies does not significantly enhance model performance. It seems that integrating introduces additional noise, complicating the predictive capability of the model. In addition to expanding the dataset, we plan to employ innovative data augmentation techniques before extracting features from EEG signals, such as using the currently popular generative adversarial networks (GANs). These methods could potentially enhance the model’s generalizability and ensure it performs well on unseen data, thus providing more reliable and accurate predictions.

Additionally, due to the focus on channel selection, connectivity features are not extracted in this study. Brain connectivity features, which describe the functional and structural connections between different regions of the brain, are crucial for understanding the complex neurodevelopmental processes in preterm infants. Future research will incorporate brain connectivity features to gain deeper insights into the neurodevelopmental changes in preterm infants. By analyzing these features, we can better understand how different brain regions interact and how these interactions evolve over time. The study on the connectivity features will provide a more holistic view of brain development and potentially reveal critical biomarkers for early diagnosis and intervention.

## 5 Conclusion

In this paper, we propose an automatic prediction framework of the FBA in preterm infants with channel selection and feature selection based on SVR model for assessing brain function maturity, in which the channel selection method is presented by combining BPSO with FA and BE methods, and the PCC-RFE-SVR feature selection method is used. Through comparative experiments, it is known that the performance of the proposed method is superior to that of other methods, particularly when selecting three-channel combination Fp1-Fp2, Fp1-T3, and Fp2-T4 and 26 features, the performance of the proposed method is best, resulting in the prediction accuracy of the FBA within ±1 week is 76.71%, and that within ±2 weeks is 94.52%.

In conclusion, this study demonstrates that effective channel selection significantly enhances the prediction accuracy of the FBA in preterm infants using EEG data. By focusing on specific channels, and utilizing advanced feature selection method, the model achieved high accuracy, reducing computational costs and overfitting risk. These findings highlight the potential of streamlined EEG analysis in clinical settings, offering a more efficient and objective tool for assessing neonatal brain development in preterm infants. Future work will aim to validate these results with larger datasets and explore additional features to further refine the predictive models.

## Data Availability

The original contributions presented in the study are included in the article/supplementary material, further inquiries can be directed to the corresponding authors.

## References

[ref1] Alotaiby T. el-Samie F. E. A. Alshebeili S. A. Ahmad I. (2015). A review of channel selection algorithms for EEG signal processing. Eurasip J. Adv. Signal Process. 2015, 1–21. doi: 10.1186/s13634-015-0251-9, PMID: 39775918

[ref2] André M. Lamblin M. D. d’Allest A. M. Curzi-Dascalova L. Moussalli-Salefranque F. Nguyen The Tich S.. (2010). Electroencephalography in premature and full-term infants. Developmental features and glossary. Neurophysiol. Clin. 40, 59–124. doi: 10.1016/j.neucli.2010.02.002, PMID: 20510792

[ref3] Baig M. Z. Aslaml N. Shum H. P. H. (2020). Filtering techniques for channel selection in motor imagery EEG applications: a survey. Artif. Intell. Rev. 53, 1207–1232. doi: 10.1007/s10462-019-09694-8

[ref4] Bhattacharyya A. Pachori R. B. (2017). A multivariate approach for patient-specific EEG seizure detection using empirical wavelet transform. IEEE Trans. Biomed. Eng. 64, 2003–2015. doi: 10.1109/TBME.2017.2650259, PMID: 28092514

[ref5] Blencowe H. Cousens S. Chou D. Oestergaard M. Say L. Moller A. B.. (2013). Born too soon: the global epidemiology of 15 million preterm births. Reprod. Health 10, 1–14. doi: 10.1186/1742-4755-10-S1-S2, PMID: 24625129 PMC3828585

[ref6] Chang C. Y. Hsu S. H. Pion-Tonachini L. Jung T. P. (2020). Evaluation of artifact subspace reconstruction for automatic artifact components removal in multi-channel EEG recordings. IEEE Trans. Biomed. Eng. 67, 1114–1121. doi: 10.1109/TBME.2019.2930186, PMID: 31329105

[ref7] de Wel O. Lavanga M. Dorado A. Jansen K. Dereymaeker A. Naulaers G.. (2017). Complexity analysis of neonatal EEG using multiscale entropy: applications in brain maturation and sleep stage classification. Entropy 19:516. doi: 10.3390/e19100516

[ref8] Dempsey E. M. Kooi E. M. W. Boylan G. (2018). It's all about the brain-neuromonitoring during newborn transition. Semin. Pediatr. Neurol. 28, 48–59. doi: 10.1016/j.spen.2018.05.006, PMID: 30522728

[ref9] Dong X. R. Kong Y. Xu Y. Zhou Y. Wang X. Xiao T.. (2021). Development and validation of auto-neo-electroencephalography (EEG) to estimate brain age and predict report conclusion for electroencephalography monitoring data in neonatal intensive care units. Anna. Transl. Med. 9:1290. doi: 10.21037/atm-21-1564, PMID: 34532427 PMC8422089

[ref10] Duun-Henriksen J. Kjaer T. W. Madsen R. E. Remvig L. S. Thomsen C. E. Sorensen H. B. D. (2012). Channel selection for automatic seizure detection. Clin. Neurophysiol. 123, 84–92. doi: 10.1016/j.clinph.2011.06.001 21752709

[ref11] Esteller R. Vachtsevanos G. Echauz J. Litt B. (2001). A comparison of waveform fractal dimension algorithms. IEEE Trans. Circ. Syst. I-Regul. Pap. 48, 177–183. doi: 10.1109/81.904882

[ref12] Fogtmann E. P. Plomgaard A. M. Greisen G. Gluud C. (2017). Prognostic accuracy of electroencephalograms in preterm infants: a systematic review. Pediatrics 139:e20161951. doi: 10.1542/peds.2016-1951, PMID: 28143915

[ref13] Gonzalez-Moreira E. Harmony T. Hinojosa-Rodríguez M. Carrillo-Prado C. Juárez-Colín M. E. Gutiérrez-Hernández C. C.. (2023). Prevention of neurological sequelae in preterm infants. Brain Sci. 13:753. doi: 10.3390/brainsci13050753, PMID: 37239225 PMC10216630

[ref14] Gschwandtner L.. (2020). Deep learning for estimation of functional brain maturation from EEG of premature neonates. Annu. Int. Conf. IEEE Eng. Med. Biol. Soc. 2020, 104–107. doi: 10.1109/EMBC44109.2020.9175380 33017941

[ref15] Guarini A. Sansavini A. Fabbri C. Savini S. Alessandroni R. Faldella G.. (2010). Long-term effects of preterm birth on language and literacy at eight years. J. Child Lang. 37, 865–885. doi: 10.1017/S0305000909990109, PMID: 19698208

[ref16] Javidan M. Yazdchi M. Baharlouei Z. Mahnam A. (2021). Feature and channel selection for designing a regression-based continuous-variable emotion recognition system with two EEG channels. Biomed. Signal Process. Control 70:102979. doi: 10.1016/j.bspc.2021.102979

[ref17] Jayakumar V. Raju R. (2010). Investigation of applications of SA in the design of dynamic cellular manufacturing systems. Int. J. Eng. Technol. 2, 220–224.

[ref18] Lee S. Soak S. Oh S. Pedrycz W. Jeon M. (2008). Modified binary particle swarm optimization. Progr. Nat. Sci. 18, 1161–1166. doi: 10.1016/j.pnsc.2008.03.018

[ref19] Li J. W. Barma S. Mak P. U. Chen F. Li C. Li M. T.. (2022). Single-Channel selection for EEG-based emotion recognition using brain rhythm sequencing. IEEE J. Biomed. Health Inform. 26, 2493–2503. doi: 10.1109/JBHI.2022.3148109, PMID: 35120013

[ref20] Mathieson S. R. Stevenson N. J. Low E. Marnane W. P. Rennie J. M. Temko A.. (2016). Validation of an automated seizure detection algorithm for term neonates. Clin. Neurophysiol. 127, 156–168. doi: 10.1016/j.clinph.2015.04.075, PMID: 26055336 PMC4727504

[ref21] Moctezuma L. A. Molinas M. (2020). Towards a minimal EEG channel array for a biometric system using resting-state and a genetic algorithm for channel selection. Sci. Rep. 10:14917. doi: 10.1038/s41598-020-72051-1, PMID: 32913275 PMC7484900

[ref22] O'Reilly D. Navakatikyan M. A. Filip M. Greene D. Van Marter L. J. (2012). Peak-to-peak amplitude in neonatal brain monitoring of premature infants. Clin. Neurophysiol. 123, 2139–2153. doi: 10.1016/j.clinph.2012.02.087, PMID: 22608473

[ref23] O'Toole J. M. Boylan G. B. Lloyd R. O. Goulding R. M. Vanhatalo S. Stevenson N. J. (2017). Detecting bursts in the EEG of very and extremely premature infants using a multi-feature approach. Med. Eng. Phys. 45, 42–50. doi: 10.1016/j.medengphy.2017.04.003, PMID: 28431822 PMC5461890

[ref24] O'Toole J. M. Boylan G. B. Vanhatalo S. Stevenson N. J. (2016). Estimating functional brain maturity in very and extremely preterm neonates using automated analysis of the electroencephalogram. Clin. Neurophysiol. 127, 2910–2918. doi: 10.1016/j.clinph.2016.02.024, PMID: 27177813

[ref25] Palmu K. Stevenson N. Wikström S. Hellström-Westas L. Vanhatalo S. Palva J. M. (2010). Optimization of an NLEO-based algorithm for automated detection of spontaneous activity transients in early preterm EEG. Physiol. Meas. 31, N85–N93. doi: 10.1088/0967-3334/31/11/N02, PMID: 20938065

[ref26] Qi F. F.. (2021). Spatiotemporal-filtering-based channel selection for single-trial EEG classification. IEEE Tran. Cybernet. 51, 558–567. doi: 10.1109/TCYB.2019.2963709, PMID: 31985451

[ref27] Ra J. S. Li T. N. Li Y. (2021). A novel permutation entropy-based EEG Channel selection for improving epileptic seizure prediction. Sensors 21:21 (23). doi: 10.3390/s21237972, PMID: 34883976 PMC8659444

[ref28] Ream M. A. Lehwald L. (2018). Neurologic consequences of preterm birth. Curr. Neurol. Neurosci. Rep. 18:48. doi: 10.1007/s11910-018-0862-2, PMID: 29907917

[ref29] Rogers C. E. Lean R. E. Wheelock M. D. Smyser C. D. (2018). Aberrant structural and functional connectivity and neurodevelopmental impairment in preterm children. J. Neurodev. Disord. 10, 1–13. doi: 10.1186/s11689-018-9253-x, PMID: 30541449 PMC6291944

[ref30] Salih A. Nichols T. Szabo L. Petersen S. E. Raisi-Estabragh Z. (2023). Conceptual overview of biological age estimation. Aging Dis. 14, 583–588. doi: 10.14336/AD.2022.1107, PMID: 37191413 PMC10187689

[ref31] Shen J. Zhang X. Huang X. Wu M. Gao J. Lu D.. (2021). An optimal channel selection for EEG-based depression detection via kernel-target alignment. IEEE J. Biomed. Health Inform. 25, 2545–2556. doi: 10.1109/JBHI.2020.3045718, PMID: 33338023

[ref32] Stevenson N. J. Oberdorfer L. Koolen N. O’Toole J. M. Werther T. Klebermass-Schrehof K.. (2017). Functional maturation in preterm infants measured by serial recording of cortical activity. Sci. Rep. 7:12969. doi: 10.1038/s41598-017-13537-3, PMID: 29021546 PMC5636845

[ref33] Stevenson N. J. Oberdorfer L. Tataranno M. L. Breakspear M. Colditz P. B. de Vries L. S.. (2020). Automated cot-side tracking of functional brain age in preterm infants. Ann. Clin. Transl. Neurol. 7, 891–902. doi: 10.1002/acn3.51043, PMID: 32368863 PMC7318094

[ref34] Toole J. M. Boylan G. B. (2017). NEURAL: quantitative features for newborn EEG using Matlab. arXiv preprint:arXiv: 1704.05694.

[ref35] Twanow J. D. E. (2017). Book review: atlas of neonatal electroencephalography. J. Child Neurol. 32:251. doi: 10.1177/0883073816675558

[ref36] Varsehi H. Firoozabadi S. M. P. (2021). An EEG channel selection method for motor imagery based brain-computer interface and neurofeedback using granger causality. Neural Netw. 133, 193–206. doi: 10.1016/j.neunet.2020.11.002, PMID: 33220643

[ref37] Webb L. Kauppila M. Roberts J. A. Vanhatalo S. Stevenson N. J. (2021). Automated detection of artefacts in neonatal EEG with residual neural networks. Comput. Methods Prog. Biomed. 208:106194. doi: 10.1016/j.cmpb.2021.106194, PMID: 34118491

[ref38] Yildirim E. Kaya Y. Kiliç F. (2021). A channel selection method for emotion recognition from EEG based on swarm-intelligence algorithms. IEEE Access 9, 109889–109902. doi: 10.1109/ACCESS.2021.3100638

[ref39] Zhang Y. T. Wang K. Wei Y. Guo X. Wen J. Luo Y. (2022). Minimal EEG channel selection for depression detection with connectivity features during sleep. Comput. Biol. Med. 147:105690. doi: 10.1016/j.compbiomed.2022.105690 35687927

